# Advanced co-culture 3D breast cancer model for investigation of fibrosis induced by external stimuli: optimization study

**DOI:** 10.1038/s41598-020-78087-7

**Published:** 2020-12-04

**Authors:** Ilya Yakavets, Aurelie Francois, Alice Benoit, Jean-Louis Merlin, Lina Bezdetnaya, Guillaume Vogin

**Affiliations:** 1 UMR7039 CRAN, Institut de Cancérologie de Lorraine, CNRS, Université de Lorraine, 6 Avenue de Bourgogne, 54519 Vandoeuvre-lès-Nancy, France; 2 UMR 7365 CNRS-UL, IMoPA, Vandœuvre-lès-Nancy, France; 3 Centre François Baclesse, Centre National de Radiothérapie du Grand-Duché du Luxembourg, Esch Sur Alzette, Luxembourg

**Keywords:** Breast cancer, Radiotherapy, Cancer models

## Abstract

Radiation-induced fibrosis (RIF) is the main late radiation toxicity in breast cancer patients. Most of the current 3D in vitro breast cancer models are composed by cancer cells only and are unable to reproduce the complex cellular homeostasis within the tumor microenvironment to study RIF mechanisms. In order to account complex cellular interactions within the tumor microenvironment, an advanced 3D spheroid model, consisting of the luminal breast cancer MCF-7 cells and MRC-5 fibroblasts, was developed. The spheroids were generated using the liquid overlay technique in culture media into 96-well plates previously coated with 1% agarose (m/v, in water). In total, 21 experimental setups were tested during the optimization of the model. The generated spheroids were characterized using fluorescence imaging, immunohistology and immunohistochemistry. The expression of ECM components was confirmed in co-culture spheroids. Using α-SMA staining, we confirmed the differentiation of healthy fibroblasts into myofibroblasts upon the co-culturing with cancer cells. The induction of fibrosis was studied in spheroids treated 24 h with 10 ng/mL TGF-β and/or 2 Gy irradiation. Overall, the developed advanced 3D stroma-rich in vitro model of breast cancer provides a possibility to study fibrosis mechanisms taking into account 3D arrangement of the complex tumor microenvironment.

## Introduction

Breast cancer is the most frequent malignancy in women worldwide^1^. In non-metastatic breast cancer, loco/regional radiotherapy (RT) is indicated in early-stage disease after breast conservation surgery and in locally advanced situations initially or post-mastectomy^2^. However, even if the radiation is delivered accordingly, up to 10% of treated patients are exposed to unexpected toxicities that may lead to sequelae^3^. Radiation activates complex signaling pathways involving inflammation and vascular damages within the irradiated microenvironement^4–6^. As a reaction, supported by the production of TGF-β by immune cells, fibroblasts tend to proliferate, migrate and transdifferentiate into myofibroblasts, which produce dysfunctional and excessive extracellular matrix (ECM) in case of deregulation^7–10^. The resulting RIF occurring 4–24 months after irradiation leads to tissue stiffening, atrophy and ultimately organ failure^11^.

The development of 3D cancer models provides both ethical and economic benefits for the prediction of tumor response to treatment (e.g., chemotherapy, RT), bridging the gap between 2D and in vivo studies, therefore reducing the number of animals sacrificed in preclinical studies^12,13^. Multicellular tumor spheroids (MCTS) are the most widely used 3D in vitro model in preclinical cancer research, reproducing important aspects of tumors, such as the presence of oxygen and nutrients gradients, and subpopulations of quiescent cells^14^. To date, several 3D breast cancer models have been applied to study post-radiation effects^15,16^. However, most of the current in vitro models are composed by only one type of cells, while to be physiologically relevant, the model should also address the complex cellular interactions within the tumor microenvironment^17^.

The aim of the present study was to develop an advanced 3D breast cancer model for the investigation of fibrosis stimulated by external stimuli, namely TGF-B and X-ray irradiation. We optimized the protocol for the formation of co-culture spheroids, consisting of the luminal breast cancer MCF-7 cells and MRC-5 fibroblasts. The development of ECM components (e.g., fibronectin, collagen) in spheroids was studied by immunohistochemistry. Finally, we explored the possibility to induce fibrosis in this newly developed co-culture MCF-7/MRC-5 spheroids by either RT or TGF-β.

## Results

### Characterization of mammary monoculture spheroids

#### Growth kinetics

**Figure 1:**
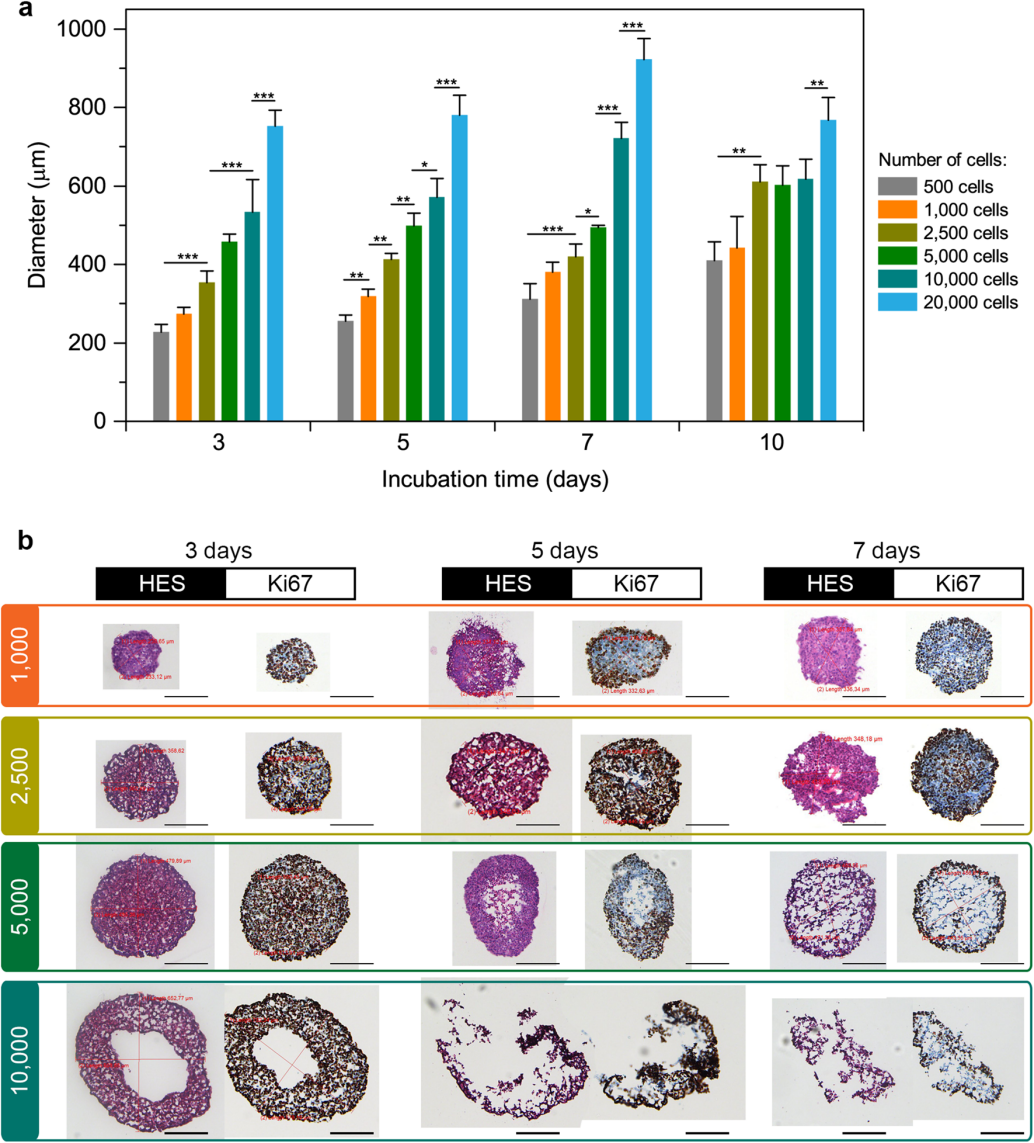
Characterization of MCF7 spheroids. **(a)** Growth kinetics. Data presented as mean ± SD [n = 3; *p < 0.05; **p < 0.01; ***p < 0.001, using ANOVA]. **(b)** Typical images of cryosections stained with HES and Ki-67 at 3, 5 and 7 days after seeding. Scale bars: 200 µm.

Monoculture 3D spheroids of MCF-7 cells, a non-invasive, hormone-positive and HER2 negative human breast cell line^18^, were successfully formed using a liquid overlay technique (LOT) in culture medium. Figure 1 displays the growth kinetics of MCF-7 spheroids in function of the initial cell density per well. We seeded from 500 to 20,000 MCF-7 cells per well and then measured spheroids diameter at 3, 5, 7 and 10 days after seeding. The size of spheroids was strongly dependent on the seeding concentration. At day 10, MCF-7 spheroids respectively presented a diameter from 420 to 800 µm, for concentrations from 500 to 20,000 cells/well. Generally, large spheroids (> 500 μm in diameter) represent the proliferation gradients of tumor cells: a necrotic core surrounded by a viable rim consisting of the layer of quiescent cells and proliferating cells on the periphery^19^. Thus, we considered that an optimal size of spheroids is about 500 µm. In fact, the spheroids formed from 500 cells/well never reached 500 µm during the whole culture period, while seeding of 20,000 cells/well resulted in 700 µm spheroids at day 3. When the spheroids grew beyond 700 μm, their size reached a plateau demonstrating the partial destruction of spheroids (single cells became visible from the outside). In total, we selected MCF-7 spheroids formed from 1000 to 10,000 cells/well for further analysis.

#### Immunohistochemical characterization

Spheroids formed from 1000 and 2500 MCF-7 cells per well were characterized by a compact core without any necrotic area during the whole growth period (Fig. 1b, lines 1–2). We also observed proliferative cells across the whole spheroids (brown Ki67 staining), with some quiescent cells at day 7 (blue color in Ki67 staining) (Fig. 1b). Spheroids initiated from 5000 cells/well displayed necrotic core after 5 days of culture. The necrosis was surrounded by quiescent cells with a following layer of proliferative cells at the periphery of spheroids. At day 7, the necrotic area was extended, taking up the bulk of the spheroid (Fig. 1b, line 3). Finally, the highest concentration of 10,000 cells/well resulted in the formation of spheroids with extensive necrosis, which was visible from day 3 after seeding (Fig. 1b, line 4). At days 5 and 7, these spheroids lost their integrity, complicating the analysis. Thus, considering criteria of expected spheroids and taking into account the addition of MRC-5 fibroblasts in co-culture model, we have selected the seeding concentrations of 1000, 2500, and 5000 cells per well for further co-culture experiments.

### Generation of co-culture mammary spheroids

Based on the optimized concentration of MCF-7 cells, we employed different culturing modalities for mixing MCF-7 cancer cells with MRC-5 fibroblasts. Co-cultured spheroids can be easily wielded by varying the seeding timing, as well as the order of the different cell types seeding^20^. In the present work, we compared simultaneous and sequential (24 h delay) seeding of different cell types in order to select the one that better represents the stroma-rich microenvironment of mammary tumors.

#### Simultaneous seeding of cancer cells and fibroblasts

**Figure 2:**
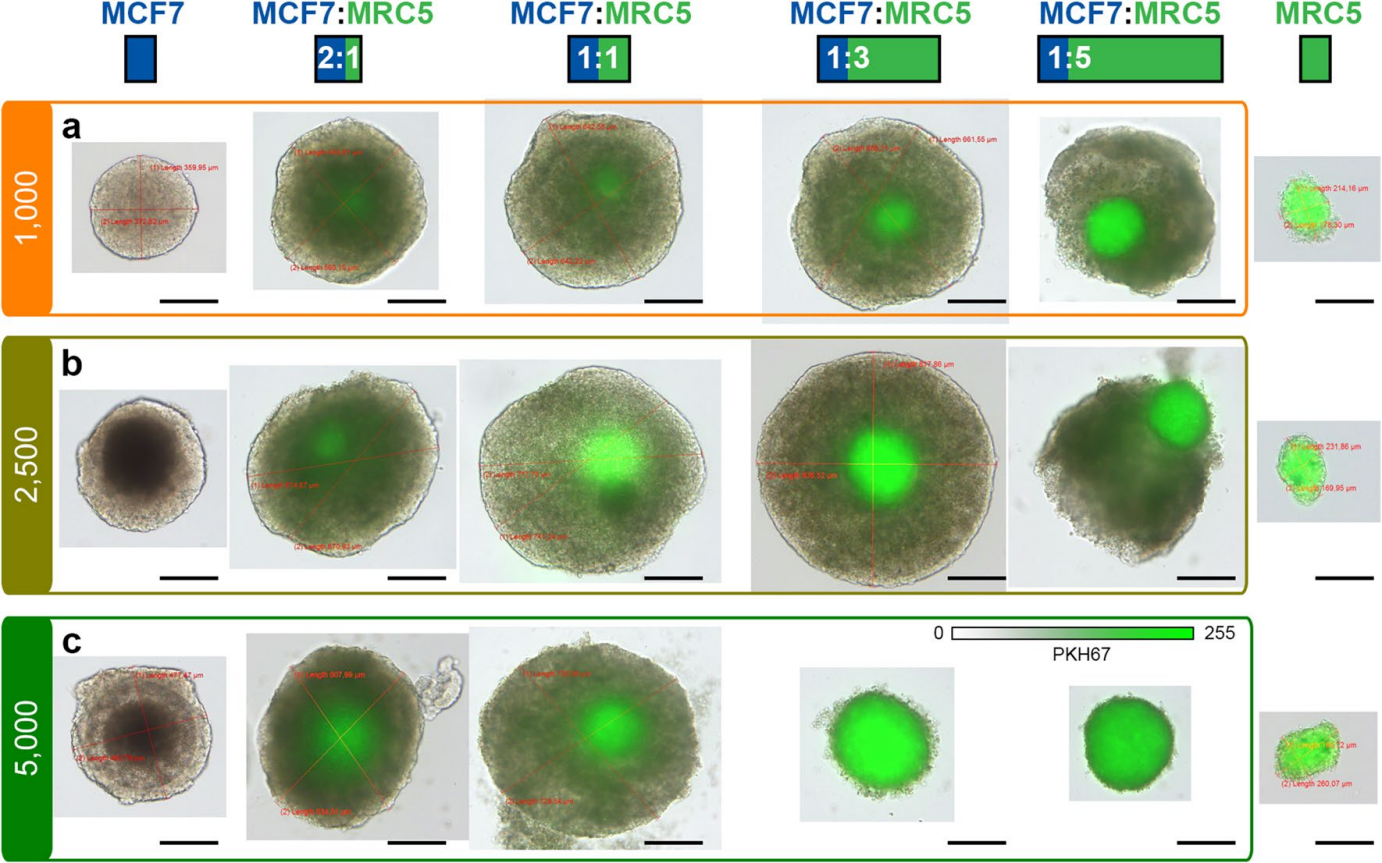
Typical optical and fluorescence images of entire co-culture MCF7/MRC5 spheroids at 7 day post-seeding. Concentration of MCF7 cells was **(a)** 1000; **(b)** 2500 and **(c)** 5000 cells per well. MRC5 monoculture fibroblasts formed from 1000; 2500 and 5000 cells per well presented in Panels **(a)–(c)**, respectively. MRC5 fibroblasts were pre-stained with green fluorescent dye (PKH67). Scale bars: 200 µm.

We have successfully generated co-culture spheroids by simultaneous seeding MCF-7 cancer cells and MRC-5 fibroblasts. In order to distinguish the fibroblasts and cancer cells in spheroids, MRC-5 fibroblasts were pre-stained with a green fluorescent membrane marker (PKH67) before seeding. We used pre-selected seeding concentrations of MCF-7 cell (1000, 2500, and 5000 cells/well) and mixed them with MRC-5 cells in different ratios, e.g., 2:1, 1:1, 1:3, and 1:5. Figure 2 represents the bright-field and fluorescence imaging of co-culture spheroids 7 days after simultaneous seeding. Notably, the distribution of MRC-5 cells (green color) in spheroids at day 5 and day 7 were similar (data not shown).

The co-culture spheroids generated with MCF-7 and MRC-5 cells had a uniform spherical shape. The decrease of MCF-7/MRC-5 ratio from 2:1 to 1:3 resulted in an increase of spheroid diameter from 576 µm up to 616 µm and from 552 µm to 828 µm for 1000 and 2500 MCF-7 cells/well, respectively. It’s worth noting that the monoculture MCF-7 spheroids generated from 1000 and 2500 cells/well reached 400 and 420 µm in size, respectively. In fact, the highest MCF-7/MRC-5 ratio 2:1 showed an imbalance between MCF-7 and MRC-5 cells, with few fibroblasts concentrated in the center of spheroids, which doesn’t reflect the clinical situation where fibroblasts are distributed more or less uniformly^21^. At the ratio MCF-7/MRC-5 1:5, the number of fibroblasts was too high, so MCF-7 cancer cells on the outer layer of the co-culture spheroids seemed to detach. These spheroids were hardly reproducible and prone to disassociate, as well as 1:3 spheroids seeded with 5,000 MCF-7 cells/well. At the highest co-culture concentrations (5,000 MCF-7 cells with 15,000 and 25,000 MRC-5 cells/well), only MRC-5 fibroblasts were visible, while the tumor cells seemed to be disassociated (Fig. 2, line 3). Of note, the monoculture MRC-5 spheroids were more compact (around 170 µm in size) and the size was independent on the cells’ seeding concentration. Such small size of spheroids along with culturing limitations of nonmalignant MRC-5 cells made hardly possible a detailed characterization of monoculture MRC-5 spheroids. Based on this, for detailed immunohistochemical characterization, we selected only 1:1 and 1:3 ratios for MCF-7 seeding concentrations of 1000 and 2500 cells/well. Table 1 summarizes the selected experimental conditions, including the seeding concentrations of MCF-7 and MRC-5 cells and proposed denotes.

**Table 1:** Conditions of cell seeding for spheroids generation.

	MCF-7 cells/well	MRC-5 cells/well	Denote
Control spheroids	1000	–	T10
	2500	–	T25
Co-culture MCF-7 : MRC-5	1000	1000 (1:1)	T10F10
	1000	3000 (1:3)	T10F30
	2500	2500 (1:1)	T25F25
	2500	7500 (1:3)	T25F75

**Figure 3:**
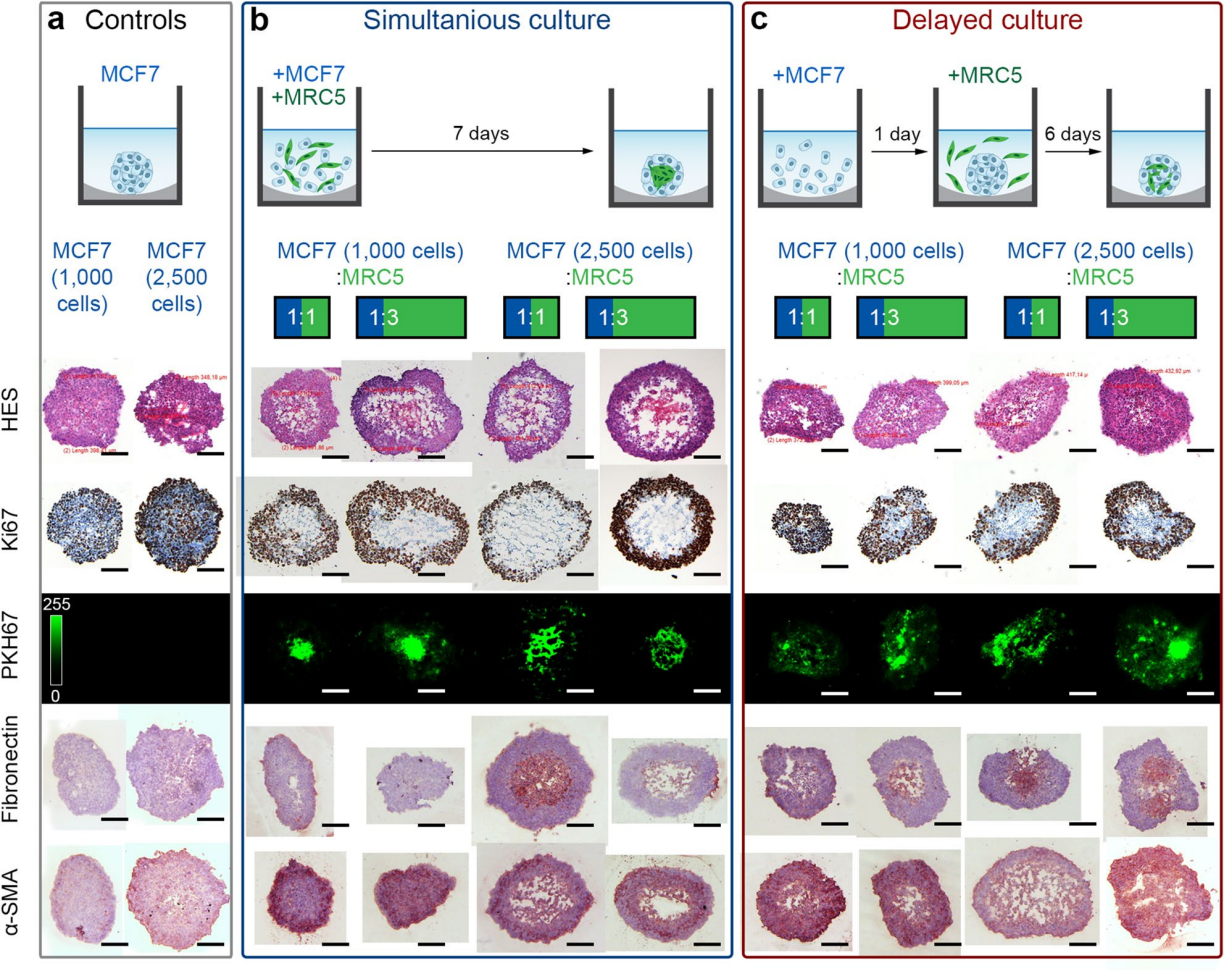
Characterization of MCF7/MCR5 co-culture spheroids initiated by **(a)** simultaneous and **(b)** sequential seeding of MRC5 cells, taken at day 7. Typical cryosections images, stained with cell viability markers (HES and Ki-67), MCR5 green fluorescence marker (PKH67), and stromal markers (*i.e.* fibronectin, α-SMA). MRC5 fibroblasts were pre-stained with green fluorescent dye (PKH67). Scale bars: 100 µm.

Figure 3 displays the detailed characterization of cryosections of monoculture (Fig. 3a) and co-culture (Fig. 3b) spheroids 7 days after seeding. In monoculture T10 and T25 spheroids, proliferative cells were detected across the whole spheroids without any necrotic area. Meanwhile, co-culture T10F10 and T10F30 spheroids (Fig. 3b) presented a core of quiescent cells surrounded by proliferating cells (Ki67 staining) with a necrotic area in the center of spheroids (Hematoxylin, Eosin and Safran (HES) staining). For T25F25 and T25F75 spheroids with an increased cell number, we observed less proliferative cells at the periphery and a larger necrotic core. In fact, the addition of MRC-5 cells accelerated the spheroids’ growth as well as the formation of the necrotic core. Overall, the co-culture spheroids met the expected criteria for different cell layers (proliferative, quiescent, and necrotic zones).

The distribution of MRC-5 cells in co-culture spheroids was visualized using fluorescence imaging (MRC-5 cells were pre-stained with green fluorescent PKH67 membrane marker). For T10F10 and T10F30 co-culture spheroids, the MRC-5 cells were mainly concentrated in the central part of the spheroid with some individual cells on the periphery. For spheroids generated from 2500 MCF-7 cells (T25F25 and T25F75), the PKH67 labeling seems to be more diffuse, perhaps due to the low cell density at the necrotic center of the spheroid. The data obtained from cryosections confirmed the results with fluorescence imaging of entire spheroids (Fig. 2).

To complete the characterization of co-culture spheroids, we assessed the expression of stromal markers such as fibronectin and α-SMA in spheroid cryosections. The fibronectin is depicted as a major constituent protein of the extracellular matrix^22–24^, and α-SMA, which is involved in motility, cell structure, and integrity, is commonly used as a marker for differentiation of fibroblasts into myofibroblasts^25,26^. As seen in Fig. 3a, fibronectin was slightly expressed in MCF-7 homospheroids (T10 and T25). In the case of T10F10 and T10F30 co-culture spheroids, only a few islets of dark brown fibronectin staining were depicted on the periphery of spheroids (Fig. 3b). The strongest fibronectin expression was evidenced in the center of T25F25 heterospheroids, while the large necrotic area in T25F75 spheroids masks the fibronectin staining. Concerning the expression of α-SMA, in monoculture MCF-7 spheroids, we observed a basal punctiform expression of α-SMA with slightly more intense expression for an increased number of cells (Fig. 3a). At the same time, for co-culture MCF-7/MRC-5 spheroids, we observed a strong expression of α-SMA (Fig. 3b). However, the expression of α-SMA was similar for spheroids with 1:1 and 1:3 ratios between MCF-7 and MRC-5 cells.

In summary, we confirmed the expression of fibronectin and α-SMA, in MCF-7/MRC-5 heterospheroids. However, simultaneous seeding resulted in the aggregation of MRC-5 fibroblasts in the center of spheroids, with successive necrosis. To avoid this situation, we tested the generation of co-culture spheroids with sequential seeding of MRC-5 fibroblasts, i.e. the fibroblasts were added to already formed MCF-7 aggregates (Fig. 3c).

#### Sequential seeding of fibroblastic cells into the co-culture spheroid model

MRC-5 cells were subsequently incubated with MCF-7 cells with a delay of 24 h or 48 h to obtain homogeneous fibroblast distribution in the co-culture spheroids, which is more representative of mammary tumors^21^. Of note, cell concentrations used were the same as those previously defined: 1000–1:1, 1000–1:3, 2500–1:1 and 2500–1:3 (number of MCF-7 cells and ratio MCF-7/MRC-5). According to obtained data, the 24 h delay resulted in more robust spheroids, which were more resistant to sampling and handling, compared to spheroids obtained with the 48 h delay (data not shown). Thus, the seeding with a delay of only 24 h was used for further experiments.

**Figure 4:**
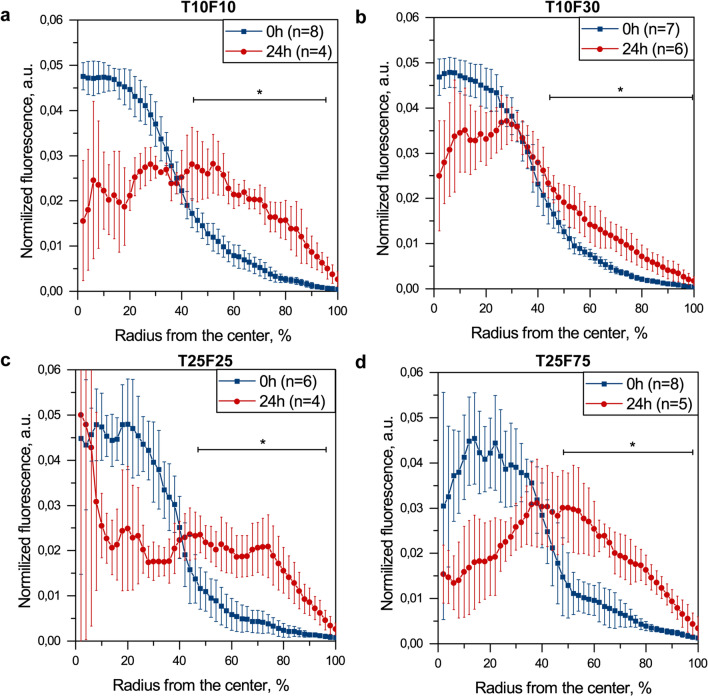
The normalized distribution profiles of PKH67 dye (MRC5 fibroblasts) across the **(a)** T10F10, **(b)** T10F30, **(c)** T25F25 and **(d)** T25F75 spheroids initiated by simultaneous (blue) and sequential (red) seeding. Data presented as mean ± SD [*p < 0.05; for each data point, using two-sample t-test].

The addition of MRC-5 fibroblasts 24 h after seeding of MCF-7 cancer cells resulted in a smaller size of spheroids and less developed necrotic area (Fig. 3c). Moreover, we observed the homogeneous distribution of clusters of PKH67 labeled MRC-5 cells across the cryosections. Finally, we confirm the overexpression of ECM markers in co-culture spheroids with delayed seeding (Fig. 3c). Importantly, there were no obvious differences in α-SMA and fibronectin expression between the co-culture spheroids using simultaneous (Fig. 3b) or delayed (Fig. 3c) cell seeding protocols. In order to compare the distribution of fibroblasts in co-culture spheroids processed either without or with 24 h delay, we quantified the normalized distribution of PKH67 fluorescence dye across the spheroids (Fig. 4). We confirmed a significantly higher fluorescence of PKH67 (*p* < 0.05; using two-sample t-test) in the peripherical regions of all types of co-culture spheroids initiated by sequential (24 h) seeding, compared to those formed simultaneously. Thus, the delayed addition of MRC-5 cells enabled the establishment of a resistant co-culture model with a homogenous distribution of fibroblasts, which reflects more precisely the clinical situation. This advanced model of breast cancer was further tested to study fibrosis induced by RT or TGF-β.

### Fibrogenesis

**Figure 5:**
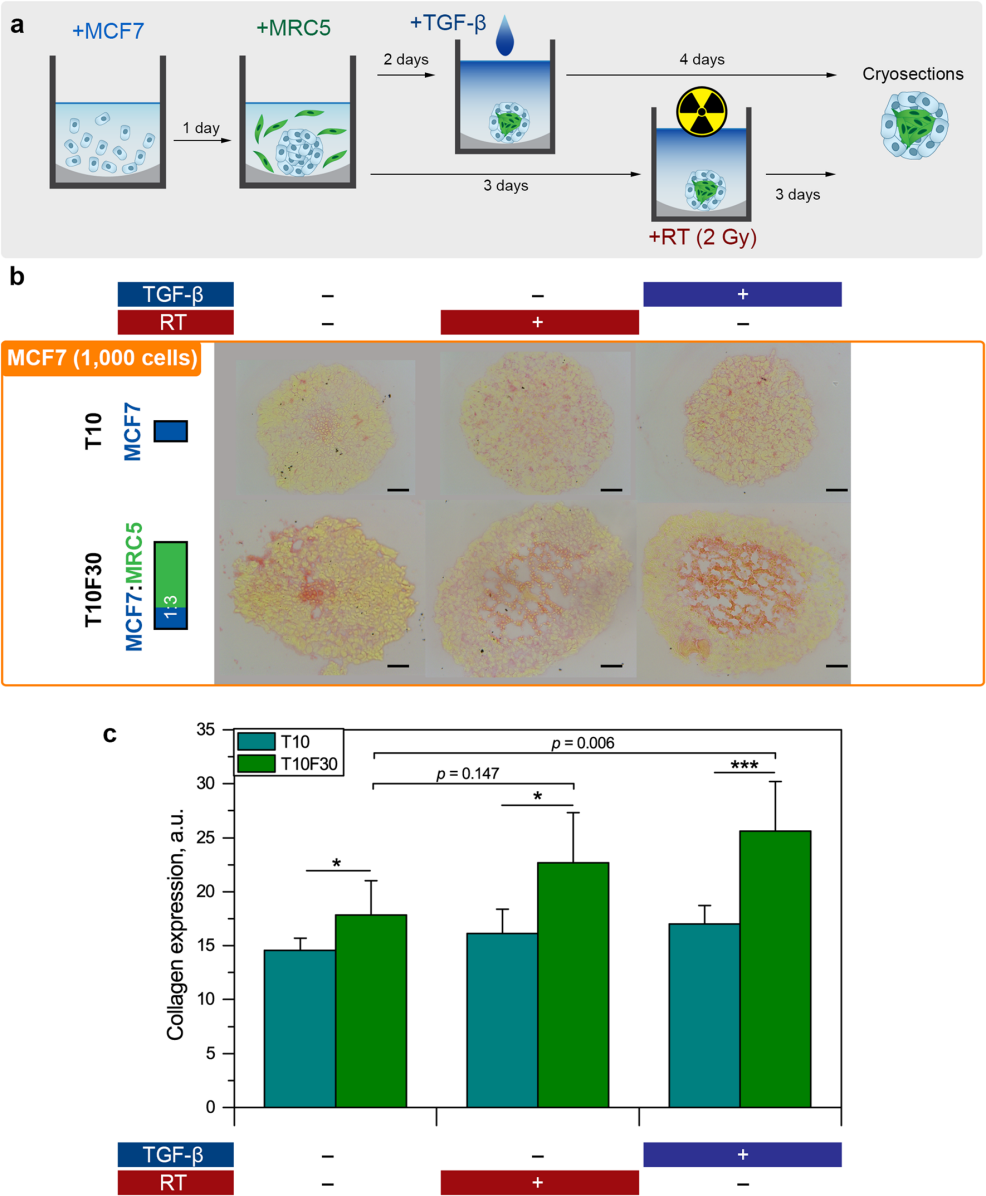
Expression of collagen in MCF7/MCR5 co-culture spheroids treated and non-treated with RT or TGF-β. **(a)** Scheme of the experiment. **(b)** Typical cryosections of T10 and T10F30 spheroids stained with Picro Sirius Red. Scale bars: 50 µm. **(c)** Cumulative quantitative analyses of collagen expression (Picro Sirius Red) in cryosections from (blue bar) monoculture T10 and (green bar) co-culture T10F30 spheroids. Data presented as mean ± SD [n = 3–10; *p < 0.05; ***p < 0.001 compared to monoculture spheroids, using two-sample t-test; ^##^*p* < 0.01 compared to control (no treatment) co-culture spheroids using ANOVA].

Picro Sirius Red staining was applied to assess the expression of collagen in spheroids subjected either to X-ray irradiation or to TGF-β (Fig. 5a). TGF-β is considered as a key molecule in the activation of the fibrotic program^27^. The cryosections after the collagen staining are presented in Fig. 5b. Moreover, the expression of collagen in spheroid cryosections was quantified and presented in Fig. 5c.

For non-treated monoculture T10 spheroids, we observed a basal collagen expression, evidenced by red staining of collagen fibers (line 1; Fig. 5b). However, there were no visual and statistical differences (*p* > 0.05; using ANOVA) observed between control and treated monoculture T10 spheroids. As anticipated, an obviously higher collagen staining (*p* = 0.044; using two-sample t-test) was displayed in non-treated co-culture T10F30 spheroids compared to monoculture T10 group (column 1; Fig. 5b). Furthermore, in co-culture spheroids subjected to 2 Gy irradiation, we observed a clear increase in collagen expression compared to the control group (17.8 ± 3.2 vs 22.7 ± 4.6, *p* = 0.147; using ANOVA), albeit not significant. Finally, the TGF-β treatment of co-culture T10F30 spheroids caused a significant collagen expression, compared to the control group (17.8 ± 3.2 vs 25.6 ± 4.6, *p* = 0.00667; using ANOVA).

## Discussion

Increasing evidence indicates that fibroblasts, particularly CAFs are critical components of the breast tumor stroma^28^. In particular, up to 80% of normal fibroblasts in breast tissue acquire the CAF phenotype during cancer progression^29^. CAFs differ from normal fibroblasts in their ability to promote tumor progression and to alter the sensitivity of cancer cells to the treatment^30^. Thus, tumor-stroma interactions should be considered in the modeling of breast cancer response to the therapies. However, the three-dimensional interactions between CAFs, breast fibroblasts and cancer cells have been poorly documented.

In the present work, we used the microwell liquid overlay technique to generate large, uniform-sized co-culture MCF-7/MRC-5 spheroids^31–33^. Co-culture breast spheroids were generated using MCF-7 tumor cells and MRC-5 fibroblasts with the successive optimization of MRC-5 seeding concentration (Fig. 2). It’s worth noting that compared to previously published protocols^33^, MCF-7 cells successfully formed uniform-sized spheroids in matrix-free culture medium without any viscosity-increasing additives such as Matrigel. Considering that 70% of the whole breast tumor volume is represented by stromal cells (e.g., CAFs)^34^, we optimized the composition and seeding procedure of the co-culture MCF-7/MRC-5 spheroids. According to the literature, stromal reaction in advanced breast carcinoma corresponds to the diffuse pattern of CAF’s localization within the tumor^35^. Thus, the sequential seeding of MRC-5 fibroblasts to the already formed MCF-7 cell aggregates (24 h delay) was considered as representative of breast tumors due to the more homogeneous distribution of MRC-5 cells within the spheroids (Fig. 4).

The CAF phenotype is responsible for altered stromal collagen remodeling and is characterized by increased expression of myofibroblast markers such as (α-SMA), vimentin, fibroblast specific protein 1 and fibroblast activating protein^36^. As was mentioned above, CAFs, which are activated from fibroblasts and overexpress α-SMA^25,26^, are considered as the main source of ECM components (e.g., fibronectin and collagen)^22–24^. We confirmed that co-culture spheroids overexpressed α-SMA, fibronectin and collagen compared to monoculture MCF-7 spheroids (Figs. 3, 5). It seems that MRC-5 fibroblasts were partially activated in co-culture with MCF-7 cancer cells. Fibroblasts, regarded as engineers of ECM, were supposed to induce the production of ECM components (i.e. fibronectin, collagen) during the co-culturing of MCF-7 with MRC-5 cells. Meanwhile, the over-activation of CAFs was anticipated by external stimuli, such as RT and TGF-β treatment. In general, our data suggest that spheroids generated after sequential seeding of fibroblasts in 1:3 ratio to the already formed MCF-7 aggregates (1000 cells/per well) were optimal for further investigation of fibrotic reactions.

Fibrosis could be activated by external (e.g., RT) or endogenous (growth factors/cytokines) stimuli^37^. The common feature of the majority of fibrotic tissues is the overexpression of TGF-β, the most potent profibrotic cytokine^27^. Radiation induces stromal production and secretion of TGF-β in the tumor microenvironment, which stimulates the production of ECM and recruits new CAFs^38^. In turn, the activated CAFs also produce ECM proteins that contribute to significant alterations of the physical properties of the stroma. Meanwhile, once activated, CAFs also produce TGF-β snowballing the fibrotic reaction^39^. Therefore, as a proof-of-the-concept, we assessed the production of collagen in the developed stroma-rich 3D model after 2-Gy single-dose RT and TGF-β treatment (Fig. 5). We observed a significant increase of collagen production in co-culture T10F30 spheroids after TGF-β treatment (*p* < 0.01). A strong trend towards increased collagen production was also noted after 2 Gy irradiation, albeit not statistically significant (*p* = 0.147). We hypothesized that in the case of 3D spheroids, RT protocol should be additionally optimized by applying higher irradiation doses or fractionated irradiation^40,41^. We also suppose that the detailed study of RIF mechanisms will require complementary experimental approaches, e.g. qRT-PCR, since the quantification of immunohistochemistry images suffers from the lack of sensitivity.

## Materials and methods

### Cell culture conditions

The MCF-7 human mammary adenocarcinoma cell line was purchased from the American Type Culture Collection (ATCC, USA) and cultured in phenol-red free Roswell Park Memorial Institute Medium-1640 (RPMI-1640, Invitrogen, USA). The culture medium was supplemented with 9% (vol/vol) heat-inactivated fetal calf serum (FCS, Sigma-Aldrich, France) and 1% (vol/vol) 0.2 M l-Glutamine (Sigma-Aldrich, France). MRC-5 human embryonic fibroblasts (ATCC, USA) were cultured in Minimum Essential Medium (MEM, Sigma-Aldrich, France) with phenol red, supplemented with 20% (vol/vol) heat-inactivated FCS, and 1% (vol/vol) of 0.1 M sodium pyruvate (Sigma-Aldrich, France). The cultures were kept at 37 ∘C, 5% of CO_2,_ and 90% humidity. The cells were reseeded once per week to ensure exponential growth at a concentration of 1 × 10^4^ cells/mL and 5 × 10^4^ cells/mL for MCF-7 and MRC-5 cells, respectively.

### Generation of homo- and hetero-spheroids

The spheroids were generated using the liquid overlay technique in 96-well plates previously coated with 1% agarose (m/v, in water). Homospheroids consisted of 1,000 to 20,000 cancer cells and were cultured at 37 ∘C, 5% CO_2_ in a humidified incubator before being taken into experiments. From 3 to 10 days of culture, MCF-7 spheroids were collected and their morphology and size were monitored by bright field microscopy (AX-70 Provis, Olympus, France).

To distinguish the different cell types within heterospheroids, fibroblasts were pre-stained with a fluorescent membrane marker PKH67 (Sigma-Aldrich, France)^42^. Briefly, MRC-5 cells were washed with serum-free medium, and a suspension of 10^7^ MRC-5 cells was incubated with 4 × 10^6^ µM of PKH67 solution for 10 min at room temperature, with gentle and regular agitation. The reaction was then stopped with the addition of two volumes of FCS. After one minute, the suspension was rinsed twice in full medium and centrifuged for 10 min at 400 *g*.

To form heterospheroids, the previously determined number of MCF-7 cells were seeded with a various number of MRC-5 fibroblasts, to reach MCF-7/MRC-5 ratios 1:1 or 1:3. Two methods of seeding were tested: either two cell types were seeded simultaneously, or fibroblasts were seeded 24 or 48 h after seeding of MCF-7 cells. This delay allowed MCF-7 cells to organize themselves into aggregates. The plates were then placed in the incubator at 37 ∘C (5% CO_2_; 90% humidity), for a period from 3 to 10 days.

### Characterization of spheroids

#### Fluorescence microscopy & immunohistological staining

Spheroids were collected and embedded into Tissue-Tek (Shandon Cryomatrix, Thermo Fisher Scientific, USA), before being frozen at − 80 ∘C. Cryosections of 10 µm thickness were made using a Cryostat. The distribution of fibroblasts, pre-stained with PKH67, within heterospheroids was observed using 460–490 nm excitation bandpass filter associated with a 505 nm dichroic mirror and 510–550 nm emission bandpass filter by means of an epifluorescence microscope (AX-70 Provis, Olympus, France).

The analysis of images was performed using ImageJ (NIH, USA) software. Custom macros for ImageJ was used to calculate the profile of dye penetration into the spheroids^43,44^. Briefly, the spheroid area was divided into 50 concentric rims with a linearly decreasing diameter. Further, the mean intensity of the pixels in each rim was calculated. After that the cumulative fluorescence was normalized for each profile. The final profiles were plotted as the mean ± standard deviation from different cryosections (n = 4–8).

For immunohistological staining, 10 µm thick cryosections were fixed with 4% paraformaldehyde for 1–3 min, then rinsed twice with water. The slides were left at room temperature until the complete drying before being stained with a solution of HES (Dako CoverStainer automate, Dako, France).

#### Immunohistochemical analyses

The immunohistochemical characterization of fixed cryosections of mono- or co-cultured spheroids was made using an automated system (Benchmark Ultra automaton, Ventana) for proliferative Ki67 marker (1:50 dilution, Monoclonal Mouse Anti-Human Ki67-Antigen, Clone MIN-1, Dako, France).

The formation of extracellular matrix and the differentiation of fibroblasts into myofibroblasts were evidenced by cryo-sections labeling with fibronectin and α-smooth muscle actin (α-SMA), respectively^42^. Endogenous peroxidase of fixed cryosections was blocked with 3% hydrogen peroxide in PBS (vol/vol) for 5 min, then the slides were washed 3 times with 0.1% tween-PBS (vol/vol, PBS-T) for 5 min. Anti-α-SMA and anti-fibronectin antibodies (Abcam, UK) diluted respectively at 1:50 and 1:100 in PBS solution of 1% (w/v) bovine serum albumin (BSA) was applied overnight at 4 °C in a humidified chamber. The slides were washed twice for 10 min in PBS-T and incubated with the secondary Biotin Goat Anti-Rabbit IgG antibody diluted 1:200 (BD Biosciences, USA) in PBS-T for 1 h at room temperature, in a humidified chamber. The slides were then rinsed twice with PBS-T, and incubated with streptavidin/peroxidase (Ready-To-Use Peroxidase Streptavidin, Vector Laboratories, USA) for 30 min in the dark. After two washings in PBS-T, the revelation was proceeded with a solution of NovaRED (Vector NovaRED Peroxidase (HRP) Substrate Kit, Vector Laboratories, USA) according to the supplier's recommendations. The slides were rinsed with distilled water before being counterstained with hematoxylin for 30 s. The slides were then dehydrated in successive baths of alcohol and xylene, and finally mounted with Pertex solution (Histolab, Sweden).

### Induction & characterization of fibrosis

#### Treatment with TGF-β and irradiation

The induction of fibrosis was studied in heterospheroids processed with delayed seeding. Spheroids were composed of either 1000 MCF-7 cells only, or 1000 MCF-7 with 3000 MRC-5 cells, i.e. the ratio cancer/normal cells was 1:3. Two days after inoculation of MRC-5 cells (i.e. 3 days after initial seeding), the culture medium was replaced by fresh serum-free RPMI/MEM (vol/vol) with or without 10 ng/mL TGF-β for 24 h.

After the 24 h treatment with TGF-β, the spheroids were subjected to irradiation at a dose of 2 Gy (6 MV photons, 200 UM/min) with a linear accelerator (Varian, USA). Control spheroids were kept without irradiation. The plates were placed in a sandwich between 1.5 cm and 2.0 cm thickness Plexiglas plates. After irradiation, a fresh serum-free culture medium, with or without 10 ng/mL TGF-β, was added. Of note, the diameter of the spheroids was controlled along the experiment in order to ensure that the depletion of serum did not induce unscheduled toxicity.

#### Collagen staining

Three days after irradiation, the spheroids were collected and embedded in Tissue-Tek before being frozen at − 80 °C. Once frozen, cryo-sections of 10 μm thickness were fixed with 4% PFA for 1–3 min, then the collagen staining was performed through Picro Sirius Red solution kit (Abcam, UK). Briefly, fixed cryo-sections were incubated with Picro Sirius Red solution for 1 h and then washed twice rapidly in acetic acid solution, supplied in the kit. The slides were then dehydrated in successive alcohol baths, and finally in xylene before being mounted with Pertex solution. In the images obtained by bright field microscopy, collagen fibers were colored in red, while the cytoplasm appeared as yellow.

Images were analyzed with Image J (NIH, USA) software. To quantify the cumulative collagen expression, the mean intensity of Picro Sirius Red staining across the cryosection area was calculated. In brief, the images were inverted, and then a green channel of RGB image was analyzed. The automatic subtraction of background was performed for all images.

### Statistics

Data were analyzed using Origin software (OriginLab, Northampton, MA, USA) and presented as the means ± standard deviation (SD). At least three independent experiments were performed. The normality of the data was checked out using the Shapiro–Wilks W test. For comparison of two groups, an unpaired, two-tailed Student’s t-test with Welch correction was used with a significance level of p < 0.05. To compare three or more groups, one-way Analysis of Variance (ANOVA) followed by Tukey’s multiple comparisons test was performed with a significance level *p* < 0.05.

## Conclusion

We have successfully developed an advanced stroma-rich 3D breast cancer spheroids consisting of the luminal breast cancer MCF-7 cells and MRC-5 fibroblasts for investigation of stomal reactions in mammary tumors. MCF-7 monoculture spheroids were generated using LOT in ECM-free culture media. Based on the optimized concentration of MCF-7 cells, we employed different co-culturing modalities for MCF-7 cancer cells with MRC-5 fibroblasts. Indeed, sequential seeding (24 h delay) of MRC-5 fibroblasts to already formed MCF-7 spheroids resulted in a homogenous distribution of fibroblasts, better reflecting the clinical situation. Co-culture spheroids overexpressed α-SMA and ECM components (e.g., fibronectin and collagen) compared to monoculture models. We demonstrated the tendency of collagen production after RT treatment of developed co-culture 3D spheroids and confirmed a strong collagen expression in spheroids exposed to TGF-β. The developed model enables the assessment of tumor tissue response to agents that mitigate or treat injury for a better understanding of the complex interaction between tumor and normal tissue in terms of fibrosis. Overall, the developed advanced 3D in vitro model of breast cancer provides a possibility to study fibrosis and optimize antifibrotic therapies in cancer treatment.

## Data Availability

The data sets used and/or analyzed during the current study are available from the corresponding author on reasonable request.
